# Gold nanoparticles as multimodality imaging agents for brain gliomas

**DOI:** 10.1186/s12951-015-0140-2

**Published:** 2015-11-20

**Authors:** Sheng-Feng Lai, Bai-Hung Ko, Chia-Chi Chien, Chia-Ju Chang, Shun-Ming Yang, Hsiang-Hsin Chen, Cyril Petibois, Dueng-Yuan Hueng, Shuk-Man Ka, Ann Chen, G. Margaritondo, Y. Hwu

**Affiliations:** Department of Engineering Science, National Cheng Kung University, Tainan, 701 Taiwan; Institute of Physics, Academia Sinica, Nankang, Taipei, 115 Taiwan; Inserm U1029 LMMA, University of Bordeaux, 33600 Pessac Cedex, France; Department of Biochemistry, School of Medicine, National Defense Medical Center, Taipei, 114 Taiwan; Department of Neurological Surgery, Tri-Service General Hospital, National Defense Medical Center, Taipei, 114 Taiwan; Institute of Aerospace and Undersea Medicine, School of Medicine, National Defense Medical Center, Taipei, 114 Taiwan; Department of Pathology, Tri-Service General Hospital, National Defense Medical Center, Taipei, 114 Taiwan; School of Basic Sciences, Ecole Polytechnique Fédérale de Lausanne (EPFL), 1015 Lausanne, Switzerland; Advanced Optoelectronic Technology Center, National Cheng Kung University, Tainan, 701 Taiwan

## Abstract

**Background:**

Nanoparticles can be used for targeted drug delivery, in particular for brain cancer therapy. However, this requires a detailed analysis of nanoparticles from the associated microvasculature to the tumor, not easy because of the required high spatial resolution. The objective of this study is to demonstrate an experimental solution of this problem, based in vivo and post-mortem whole organ imaging plus nanoscale 3-dimensional (3D) X-ray microscopy.

**Results:**

The use of gold nanoparticles (AuNPs) as contrast agents paved the way to a detailed high-resolution three dimensional (3D) X-ray and fluorescence imaging analysis of the relation between xenografted glioma cells and the tumor-induced angiogenic microvasculature. The images of the angiogenic microvessels revealed nanoparticle leakage. Complementary tests showed that after endocytotic internalization fluorescent AuNPs allow the visible-light detection of cells.

**Conclusions:**

AuNP-loading of cells could be extended from the case presented here to other imaging techniques. In our study, they enabled us to (1) identify primary glioma cells at inoculation sites in mice brains; (2) follow the subsequent development of gliomas. (3) Detect the full details of the tumor-related microvasculature; (4) Finding leakage of AuNPs from the tumor-related vasculature, in contrast to no leakage from normal vasculature.

**Electronic supplementary material:**

The online version of this article (doi:10.1186/s12951-015-0140-2) contains supplementary material, which is available to authorized users.

## Background

Along the path towards the potential use of nanoparticles as drug carriers for cancer therapy and diagnosis [1–3], some critical issues are related to the tumor-microvasculature leakage of nanoparticles [4–6]. This leakage could notably impact drug carrying nanotechnologies [7–10]. Observing nanoparticle leakage, however, is a challenge for most imaging methods. The objective of our study was to practically demonstrate a solution to this problem in the specific case of glioma tumors, one of highest mortality rate and difficult to treat cancers. Glioma is also one type of tumor expresses high level of angiogenesis [11].

In a broader picture, the above problem is related to the issue of the BBB (blood brain barrier) breakdown [12, 13]. A protective mechanism for non-reproducible neurons from most macromolecules, BBB also impedes the delivery of therapeutic agents to specific region of the brain and therefore is an obstacle in the treatments of many brain disorders. In the case of brain tumor treatment, BBB is not always intact due to the abnormal structure of the vascular endothelial cells and the associated pericytes: this could create therapeutic opportunities [14–16].

However, many questions about the corresponding mechanisms remain to be clarified by suitable experiments. This creates the need for new imaging approaches that should complement well-established techniques such as immunochemical methods, e.g., anti-IgG immunohistology [17, 18]. Ideally, the new approaches should detect all the details of the leakage of nanoparticles from the microvasculature into the surrounding tumor tissues.

This requires simultaneous imaging of angiogenesis vessels and nanoparticles. Furthermore, the imaging should be in 3D to link the particles outside the vessels with the vessel leakage. Tumor angiogenesis vessels were previously detected with techniques such as magnetic resonance imaging (MRI) [19, 20] and ultrasound imaging [21], whose resolution, however, does not distinguish perfused nanoparticles from those leaked from angiogenic blood vessels.

X-ray imaging is a natural candidate for this task, since recent progress brought it close to nanometer-level resolution; furthermore, it was successfully tested for complete profiling of the tumor angiogenesis microvasculature [22]. Our objective here is to practically demonstrate its use to detect nanoparticle leakage.

## Results and discussion

Our study is based on multiple non-conventional imaging techniques: phase contrast microradiology [23, 24], transmission X-ray microscopy (TXM) [25–29] and visible fluorescent nanoparticle imaging. The corresponding performances were augmented by gold nanoparticles (AuNPs), which were already used for X-ray contrast enhancement [22, 30–34].

We investigated both bare and 11-MUA-coated AuNPs (MUA = mercapto − undecanoicacid). Note that AuNPs become photoluminescent at very small sizes, creating the opportunity to combine X-ray imaging with fluorescent microscopy [35–37]—a strategy that is part of our present work.

This combined approach included two different applications: first, after loading large AuNP amounts in glioma cells and inoculating them, we traced the tumor development. Specifically, we could detect and analyze the corresponding anomalous microvasculature, down to very small (a few µm) vessels. Second, we could study the leakage of AuNPs from microvessels using in parallel both X-ray microscopy and fluorescence microscopy.

Our experiments were based on large loads of AuNPs in glioma cells, up to >50 pg/cell. We had previously found that large amounts of AuNPs can be internalized in cells via endocytosis without affecting their viability [38–42]. In the present case, we observed that the AuNPs, even at our highest load levels, do not affect the proliferation and other functions of glioma cells. They do provide, however, the required X-ray contrast enhancement for detailed monitoring of the tumor growth. We could thus specifically analyze the relation between the primary inoculated cancer cells, their proliferation and the subsequent tumor growth and metastasis.

The tumor-related microvasculature is of course a crucial issue in this analysis. For this, in addition to AuNPs, we also used as contrast agent high-density BaSO_4_ nanoparticle colloid, following in both cases the nanoparticle flow in the blood system [22]. The analysis of microvasculature was also performed in 3D by X-ray microtomography, reaching ~1 µm spatial resolution.

The X-ray imaging methods included in specific cases 20 nm resolution TXM [25–29], reaching ~20 nm resolution. In the analysis of features of particular interest this revealed, for example, individual cells adhering to the vessel walls away from the inoculation site—indicating migration along the vessel system.

Prior to the imaging experiments, we performed viability tests. In general, the cell survival rate was higher after exposure to bare AuNPs than to MUA-AuNPs. The concentration of bare AuNPs reached 2 mM without interfering with the cell viability (Additional file 1: Figure S1a), whereas effects were detected at 0.5 mM concentration in the case of MUA-AuNPs. Furthermore, we found that the GBM8401 cell line is less tolerant than the U87 cell line, with detectable viability effects at a concentration of 0.5 mM (Additional file 1: Figure S1b).

Optical microscopy images revealed large amount of bare AuNPs in the U87 cells (Additional file 1: Figure S1c, first row). On the contrary, the uptake of bare AuNPs in GBM8401 was relative low and the cell morphology appeared altered (Additional file 1: Figure S1c, second row).

Fluorescence images of U87 cells loaded with MUA-AuNPs exhibited bright areas revealing a high amount of internalized nanoparticles. TEM images and histopathology optical micrographs confirmed the internalization of bare AuNPs and MUA-AuNPs (Additional file 1: Figure S2).

The initial part of our study used microresolution radiography with bare AuNPs as biomarkers to localize in vivo tumor cells in the brain. In addition, fluorescence microscopy tests used MUA-AuNPs. We observed tumor cells growing into gliomas without interference from the AuNPs.

**Fig. 1:**
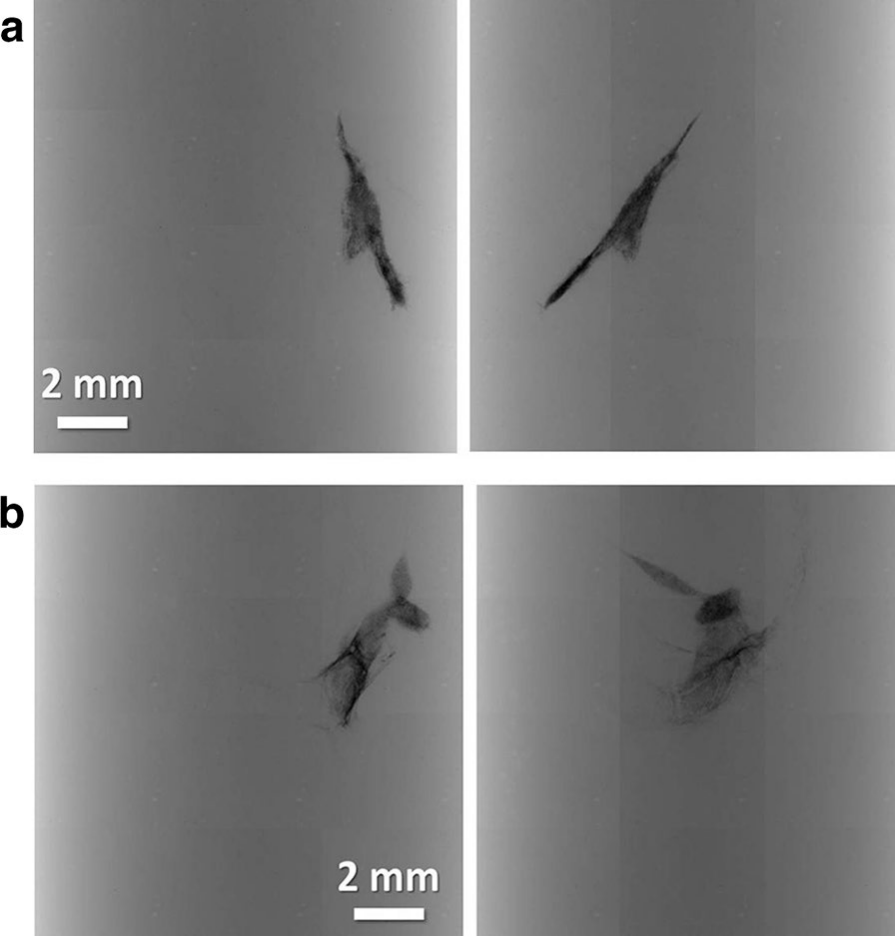
Projection x-ray images of tumor cells loaded with bare AuNPs in the brain, from two examples (**a** and **b**) each of two different points of views: anteroposterior and lateral. The black dots correspond to tumor cells

**Fig. 2:**
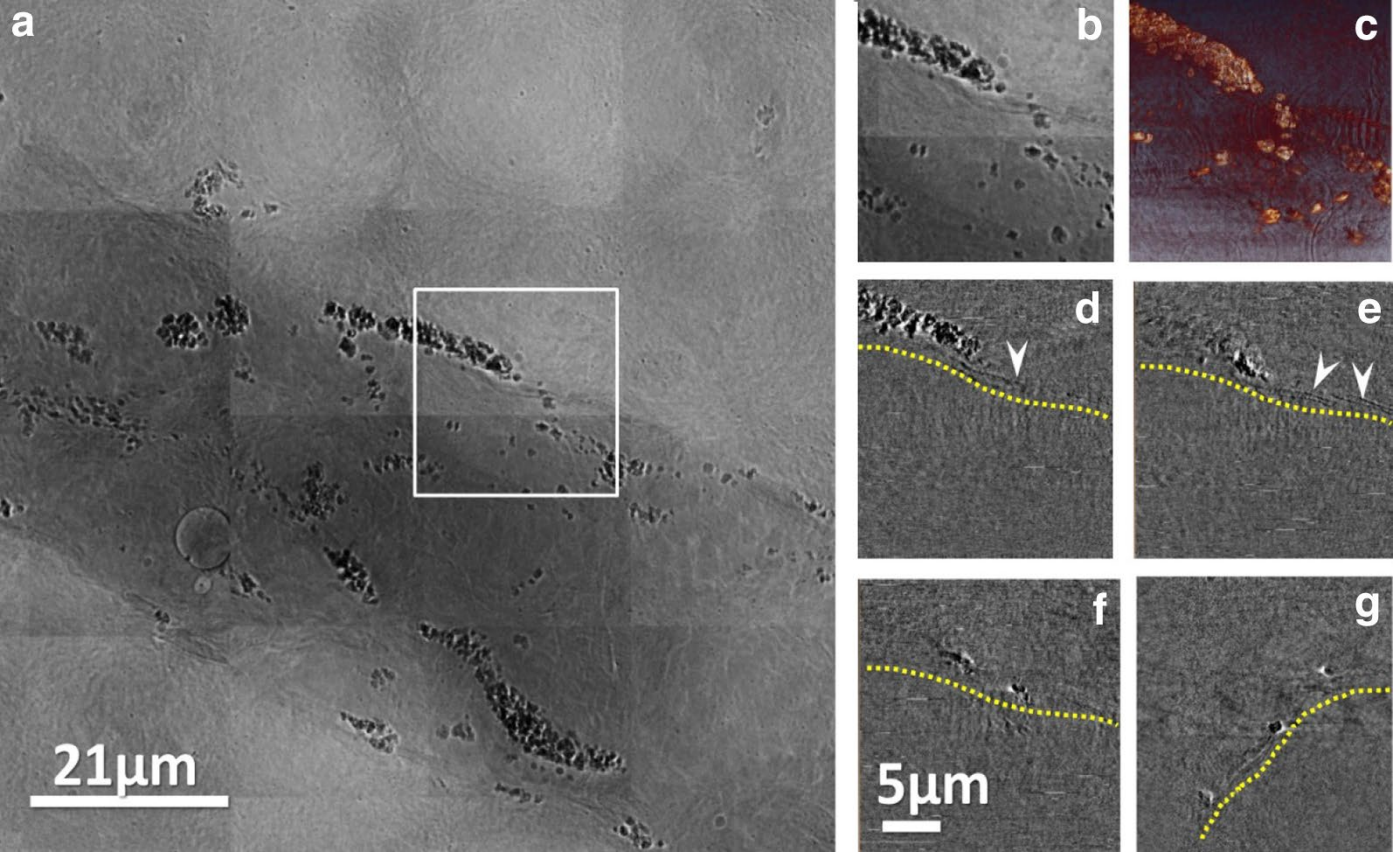
TXM micrographs of AuNP loaded U87 cells in brain tissues. TXM micrographs of bare AuNPs in U87 cells in brain tissues. The *dark grey* regions outline small vessels, whereas the *black dots* correspond to bare AuNPs, which are internalized by the U87 cells. **a** Patchwork of projection images on a region showing a microvessel with U87 cells that adhere to its wall, **b** individual projection image, **c** 3D tomographic reconstructed image and **d**–**g** tomographic reconstructed images of slices; the internal vessels are below the *yellow line*. Cells with bare AuNPs are seen on the surface of vessels (above the *yellow lines*)

The perfused cells are visible as black dots along the injection needle paths in the radiographs of Fig. 1. We see here two examples (Fig. 1a, b) of projection images each with two different viewing directions; complete tomography reconstruction to reveal the detailed 3D tumor micro-environment. Most tumor cells near the injection site are found on the surfaces of small vessels (Fig. 2). In Fig. 2a, b, such vessels correspond to the central dark grey area and the tumor cells are the black dots in the brain tissue.

The reconstructed tomographic images of Figs 2d–g correspond to the marked region in Fig. 2b, and reveal that the primary tumor cells did not invade the vessels besides adhering to their surfaces. TEM did confirm the presence of tumor cells with bare AuNPs (Additional file 1: Figure S3).

**Fig. 3:**
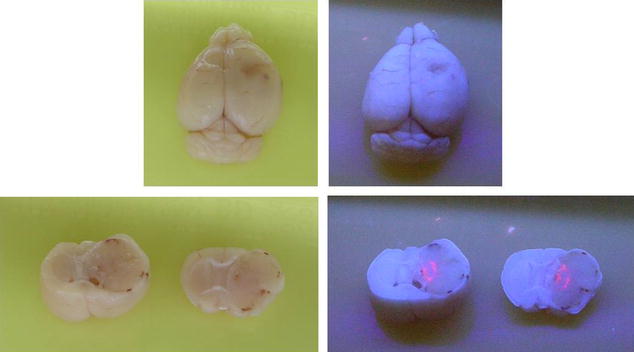
Optical images of entire brain with MUA-AuNPs. Optical images of the entire brain reveal tumor cells with fluorescent MUA-AuNPs. The images on the left-hand side were taken under normal illumination and show no fluorescence. The right-hand images were taken under UV light exposure and reveal the nanoparticle fluorescence

Fluorescent micrographs of UV-stimulated MUA-AuNP specimens confirmed the above results. Furthermore, they also revealed gliomas in the brain caused by the injected cells. Figure 3 shows primary tumor cells in brain tumor tissues; TEM images (Additional file 1: Figure S4) and histopathology micrographs (Additional file 1: Figure S2d) corroborate these observations.

**Fig. 4:**
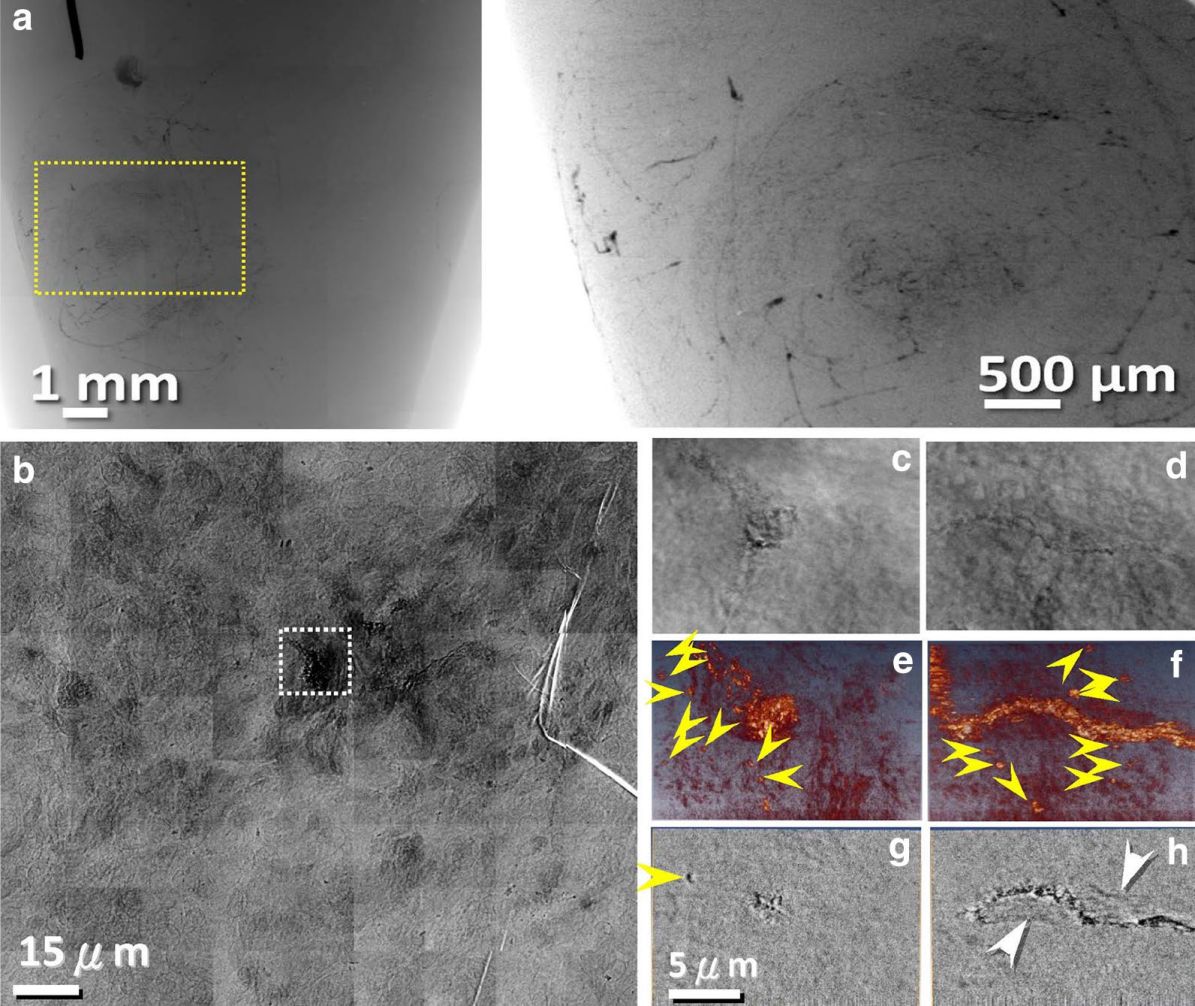
X-ray micrographs show leakage of AuNPs out of vessels. X-ray micrographs show bare AuNPs leaking out of vessels in the proximity of brain tumors. **a** The projection X-ray images at two different magnifications of a U87 mouse brain tumor with bare AuNPs perfusion show the whole brain vasculature, **b** patchwork of TXM images; **c**, **d** individual TXM projection images taken at different angles, corresponding to the *light-grey rectangle* in (**b**); **e**, **f** 3D tomographic reconstructed images, corresponding to the (**c**, **d**) images; **g**, **h** tomographic reconstructed single-slice images of (**e**, **f**). The *yellow arrowheads* mark bare AuNPs leaking out of tumor vessels. The *white arrows* mark the nuclei of endothelial cells

**Fig. 5:**
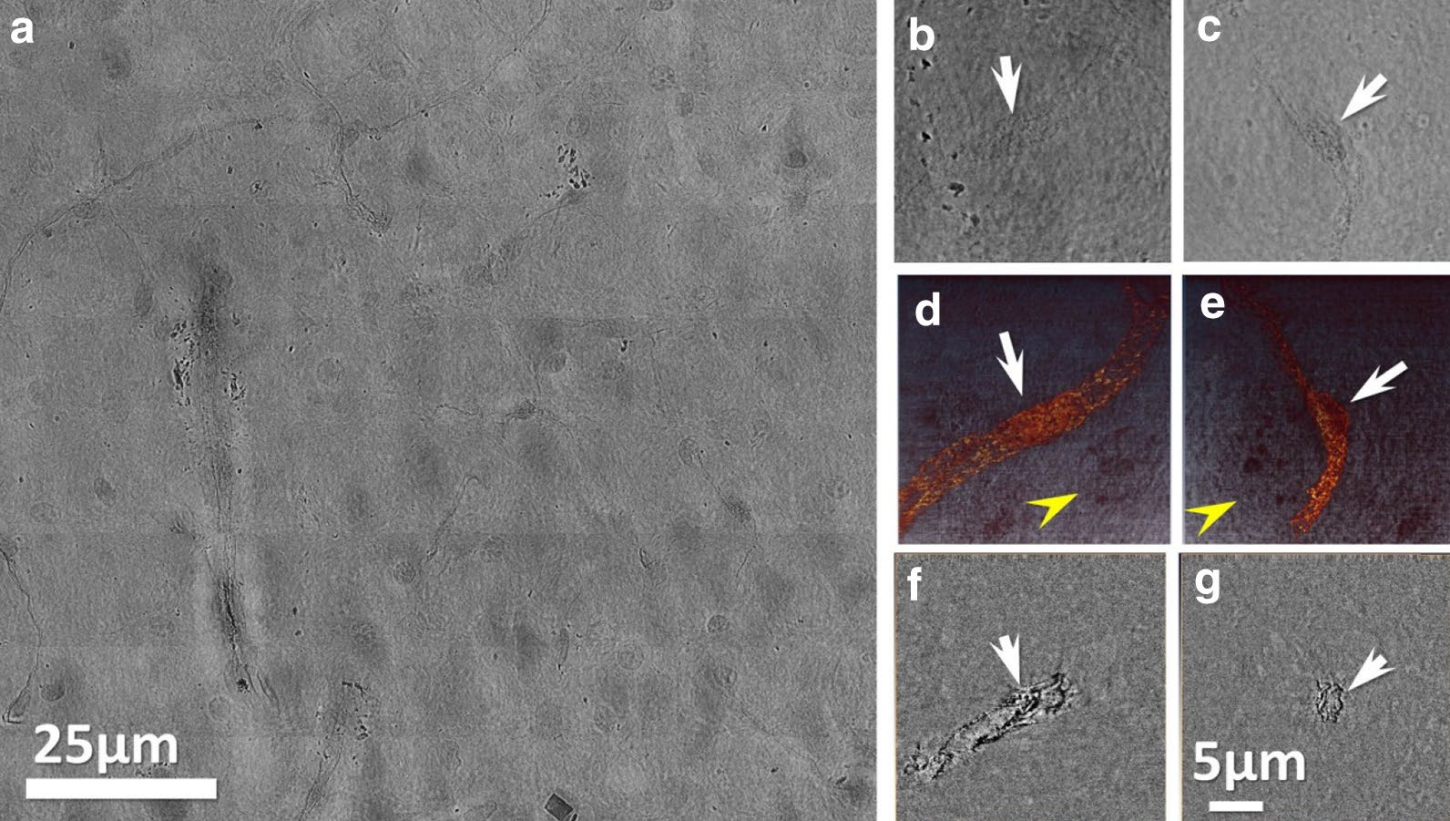
TXM micrographs show no leakage of AuNPs of normal brain vessels. TXM images of bare AuNPs in normal brain vessels. **a** Patchwork of projection images; **b**, **c** individual projection images taken at different angles; **d**, **e** 3D tomographic reconstructed images, corresponding to (**b**, **c**) and (**f**, **g**) tomographic reconstructed single-slice images of (**d**, **e**). *Yellow arrowheads* mark the nuclei of brain cells. *White arrows* mark the nuclei of endothelial cells

Finally, we would like to focus on the key result of our study: the leakage of nanoparticles from the microvasculature into the tumor tissue. Typical results are shown in Fig. 4. There we see high-resolution TXM images and corresponding tomographic reconstructions. Such images clearly reveal the leakage of AuNPs—specifically, bare nanoparticles. More specifically, leakage is observed for the tumor areas, whereas we detect no leakage out of vessels in the areas of healthy brain tissues (Fig. 5). To the best of our knowledge, this is the first direct imaging of the leakage of metal nanoparticles to tumors in the brain.

We did not find any major limitations in the use of our approach, in particular in the multiple advantages of inoculating AuNPs-loaded cancer cells. This raises the question of the potential applications of this approach to other imaging techniques. The key problem is to reach a sufficient concentration without affecting the cancer cell activity. AuNPs are ideal for that because of their high biocompatibility: they could be used for imaging techniques such as infrared spectromicroscopy [11] that can easily detect them over the background. AuNPs based on gold radioisotopes could assist techniques such as Single Photon Emission Computer Tomography (SPECT) [43], again taking advantage of the biocompatibility and high loading levels.

For medical imaging techniques such as MRI [40, 44], positron emission tomography (PET) [45], SPECT [46], computed tomography (CT) [34] and ultrasound imaging [21], many different nanoparticles were reported to enhance the sensitivity. Along this line, nanoparticles can be loaded into cancer cells for detection in vivo in animal models. In vivo imaging is not likely to reach the resolution required to detect single cells, but can identify tumor locations and possibly metastasis.

The high resolution achieved with X-ray microscopy is critical for the detection of nanoparticle leakage, particularly at the early stage of the BBB breakdown. The low concentration of nanoparticles leaking from blood vessels could limit the applications to animal models and in general to drug delivery. However, it should still be possible to use nanoparticles as contrast agents for the detection of the location and outline of tumors in medical diagnostics. Furthermore, suitable nanoparticle modifications could allow the in vivo detection of leakage for diagnostics.

## Conclusions

These are the main results of our study: first, by loading glioma cells with high concentrations of bare AuNPs or MUA-AuNPs we made them detectable by X-ray and fluorescence imaging. Second, we could thus identify primary glioma cells at inoculation sites in mice brains. Third, we could then follow the subsequent development of gliomas. Fourth, we detected the full details of the tumor-related microvasculature. Fifth, we found leakage of AuNPs from the tumor-related vasculature, in contrast to no leakage from normal vasculature.

The significance of our results goes beyond the mere tests of the experimental strategy. By showing that we can detect leakage of nanoparticles into brain tumor areas, we open the door to a much-needed analysis of a phenomenon of great importance, in particular because of its role in nanomedicine therapies for brain diseases.

## Methods

### Cell viability tests

U87 and GBM8401 glioma cell lines were incubated with Dulbecco’s Modified Eagle’s Medium (DMEM)/10 % fetal bovine serum (FBS) and cultured at 37 C in humid air with 5 % CO_2_. U87 and GBM8401 glioma cell lines were separately co-cultured with 0, 0.5, 1, 1.5 and 2 mm of bare Au NPs and MUA-Au NPs for 24 h to test the viability—which was quantitatively assessed by counting the number of living cells using the ADAM cell counter (BioAssay Systems, Hayward, CA, USA). The nanoparticle preparation is described, for example, in Ref. [47, 48].

### In vivo orthotopic xenografting

All procedures involving experimental animals in this work were approved by the Academia Sinica Institutional Animal Care and Utilization Committee (AS IACUC). BALB/cAnNCg-Foxn1nu/CrlNarl nude mice were purchased from the National Laboratory Animal Center, Taiwan. The mice were housed in individual ventilated cages (five per cage) with wood chip bedding and kept at 24 ± 2 °C with a humidity of 40–70 % and a 12-hour light/dark cycle.

U87 glioma cell lines were co-cultured with 2 mm bare-AuNPs or 0.5 µm MUA-AuNPs for 24 h. The method for in vivo orthotopic xenografting was previously described [42, 49]. In brief, cells were harvested and re-suspended in PBS. A total of 5 × 10^5^ U87 cells in 5 μl PBS-containing glioma cells suspension solution were inoculated via a 32 gauge needle (injection rate 1 μl per min) into the basal ganglia of the right brain hemisphere. Tumor imaging started after either 7 or 14 days.

### Contrast agent perfusion for microvessel imaging

After a midline neck incision, the right common carotid artery (CCA) was isolated and a small incision was made. A catheter (PE-08, BB31695, Scientific Commodities, Inc., ID 0.2 mm, OD 0.36 mm) was inserted through it. 6–0 silk sutures were tightly tied around the vessel. The tubing was secured with two knots around the CCA and then perfused with 500 µL heparinized (500 U/mL) bare-AuNPs (1.57 mg/mL concentration).

### In vivo X-ray imaging

Microradiology was implemented with unmonochromatized (white) synchrotron X-rays emitted by the 01-A beamline wavelength shifter of the National Synchrotron Radiation Research Center (Taiwan). The photon energy ranged from 4–30 keV with a peak intensity at ~12 keV; the beam current was kept constant at 360 mA with the top-up operation mode.

We obtained 4.5 × 3.4 mm^2^ radiographs by converting the X-rays into visible light using a CdWO_4_ single crystal scintillator and then detecting the photons with an optical microscope equipped with a 1600 × 1200 pixel CCD camera (model 211, Diagnostic Instruments). We reduced the radiation dose by attenuating the X-ray beam with two 550 µm silicon wafers.

The typical exposure time was ~100 ms with and the dosage 33.9 Gy. The sample-scintillator distance was 5 cm. We used a 2 × lens in the optical microscope to obtain the desired field of view; the pixel size in the final image was 2.8 × 2.8 µm^2^.

### Tissue sample preparation

After developing glioma, the mice were sacrificed by an overdose of Zoletil 50 (50 mg/kg; Virbac Laboratories, Carros, France) administered by intramuscular injection (weight ~20–25 g). Brain tissue specimens were immersed in 3.7 % paraformaldehyde for 24 h. After fixation, the tissues were washed by PBS (phosphate buffer solution) three times for 1 h. Before embedding, the tissues were separated in two groups, one for X-ray imaging (and in some cases fluorescence microscopy) with micron-level resolution and the other for nanoresolution X-ray microscopy.

Microresolution X-ray imaging was performed on fresh thick tissue specimens in 3.7 % paraformaldehyde. Nanoresolution imaging was performed instead on specimens embedded in paraffin. Before being embedded, all tissues were dehydrated by sequential immersions in ethanol, from low to high concentrations.

The specimens for nanoresolution were sliced to 30 µm thickness and immersed in Xylene three times for 5 min, to remove the remaining wax. Then, they were dehydrated with the procedure described above and immersed in distilled water. The specimens were then processed with heavy metal staining (osmium), washed with distilled water three times for 5 min, dehydrated and embedded in Embed-812 Resin (EMS, Hatfield, PA). We also followed this procedure to prepare specimens for standard visible microscopy analysis, except that the heavy metal staining was replaced by H&E staining.

### Tomography

Thick samples in resin were used to take sets of 1000 images at equal angular distances within 180 degrees. The exposure time was 100 ms per image, corresponding to a 33.9 Gy dose. The tomographic reconstruction was performed with the IDL software. All reconstructed images were processed with the Amira software to obtain 3D renditions.

### X-ray microscopy with nanoscale resolution

These tests were performed on the 32-ID microscopy beamline of the advanced photon source (APS) at the Argonne National Laboratory. Our full-field TXM there uses a set of capillary condensers to precisely illuminate the object with a variable numerical aperture adjusted to match the specific FZP objective. The condensers are elliptically shaped glass capillaries. The inner diameter of 0.9 mm was chosen to maximize the vertical acceptance of the APS undulator beam at 65 m from the source.

The estimated monochromatic X-ray flux through a Si (111) double crystal monochromator focused by the condenser was 2 × 10^11^ photons/s at 8 keV. The high brightness of the APS and the optimized condensers design yielded an excellent imaging rate of 50 ms/frame with ~1 × 10^4^ CCD counts per pixel. We systematically implemented phase contrast imaging using with an Au Zernike phase ring placed at the back focal plane of the FZP objective.

## Additional file

10.1186/s12951-015-0140-2 Cell viability analysis. U87 and GBM8401 glioma cell lines were separately co-cultured with 0, 0.5, 1, 1.5 and 2 mM of bare Au NPs (a) and MUA-Au NPs (b) for 24 hr, U87 and GM18401 cell lines showed a good biocompatibility of bare-AuNPs. However, the biocompatibility was slightly lower for MUA-AuNPs than for bare-AuNPs. (c) Optical images of bare AuNPs co-cultured with U87 and GBM 8401 cell lines. The optical images and fluorescence imaging of MUA-AuNPs co-cultured with U87 (d), and GBM 8401 (e) cell lines. **Figure S2.** Transmission electron microscope images of U87 cell in different conditions. (a) Control specimen, without any treatment. (b) and (c) Bare AuNPs MUA-AuNPs co-cultured with cells. (d) In vivo cellular tracking U87 glioma cells loaded with bare AuNPs demonstrated invasive glioma cells in brain. **Figure S3.** Transmission electron microscope images of tumor cells with bare-AuNPs in brain tumor tissue. Scale bars: 1 μm. **Figure S4.** Transmission electron microscope images of tumor cells with MUA-AuNPs in brain tumor tissue. Scale bars: 0.5 μm.
