# Supplementary material for: Gold nanoparticles as multimodality imaging agents for brain gliomas
Source: J Nanobiotechnology. 2015 Nov 20;13:85. doi: 10.1186/s12951-015-0140-2 (PMC4654925; doi:10.1186/s12951-015-0140-2)
Supplement: Supplementary file 1 — 10.1186/s12951-015-0140-2 Cell viability analysis. U87 and GBM8401 glioma cell lines were separately co-cultured with 0, 0.5, 1, 1.5 and 2 mM of bare Au NPs (a) and MUA-Au NPs (b) for 24 hr, U87 and GM18401 cell lines showed a good biocompatibility of bare-AuNPs. However, the biocompatibility was slightly lower for MUA-AuNPs than for bare-AuNPs. (c) Optical images of bare AuNPs co-cultured with U87 and GBM 8401 cell lines. The optical images and fluorescence imaging of MUA-AuNPs co-cultured with U87 (d), and GBM 8401 (e) cell lines. Figure S2. Transmission electron microscope images of U87 cell in different conditions. (a) Control specimen, without any treatment. (b) and (c) Bare AuNPs MUA-AuNPs co-cultured with cells. (d) In vivo cellular tracking U87 glioma cells loaded with bare AuNPs demonstrated invasive glioma cells in brain. Figure S3. Transmission electron microscope images of tumor cells with bare-AuNPs in brain tumor tissue. Scale bars: 1 μm. Figure S4. Transmission electron microscope images of tumor cells with MUA-AuNPs in brain tumor tissue. Scale bars: 0.5 μm. [file 12951_2015_140_MOESM1_ESM.docx]

**Supplementary Information**


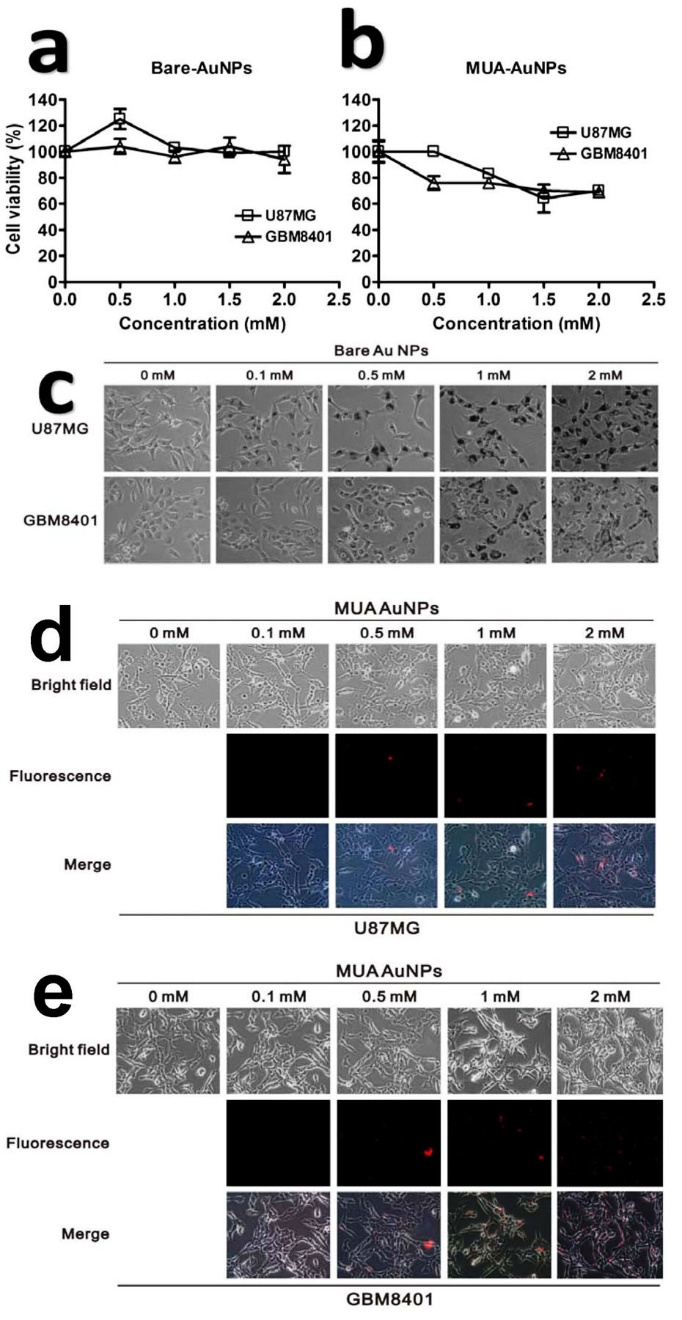


**Figure S1 -** Cell viability analysis. U87 and GBM8401 glioma cell lines were separately co-cultured with 0, 0.5, 1, 1.5 and 2 mM of bare Au NPs (a) and MUA-Au NPs (b) for 24 hr, U87 and GM18401 cell lines showed a good biocompatibility of bare-AuNPs. However, the biocompatibility was slightly lower for MUA-AuNPs than for bare-AuNPs. (c) Optical images of bare AuNPs co-cultured with U87 and GBM 8401 cell lines. The optical images and fluorescence imaging of MUA-AuNPs co-cultured with U87 (d), and GBM 8401 (e) cell lines.


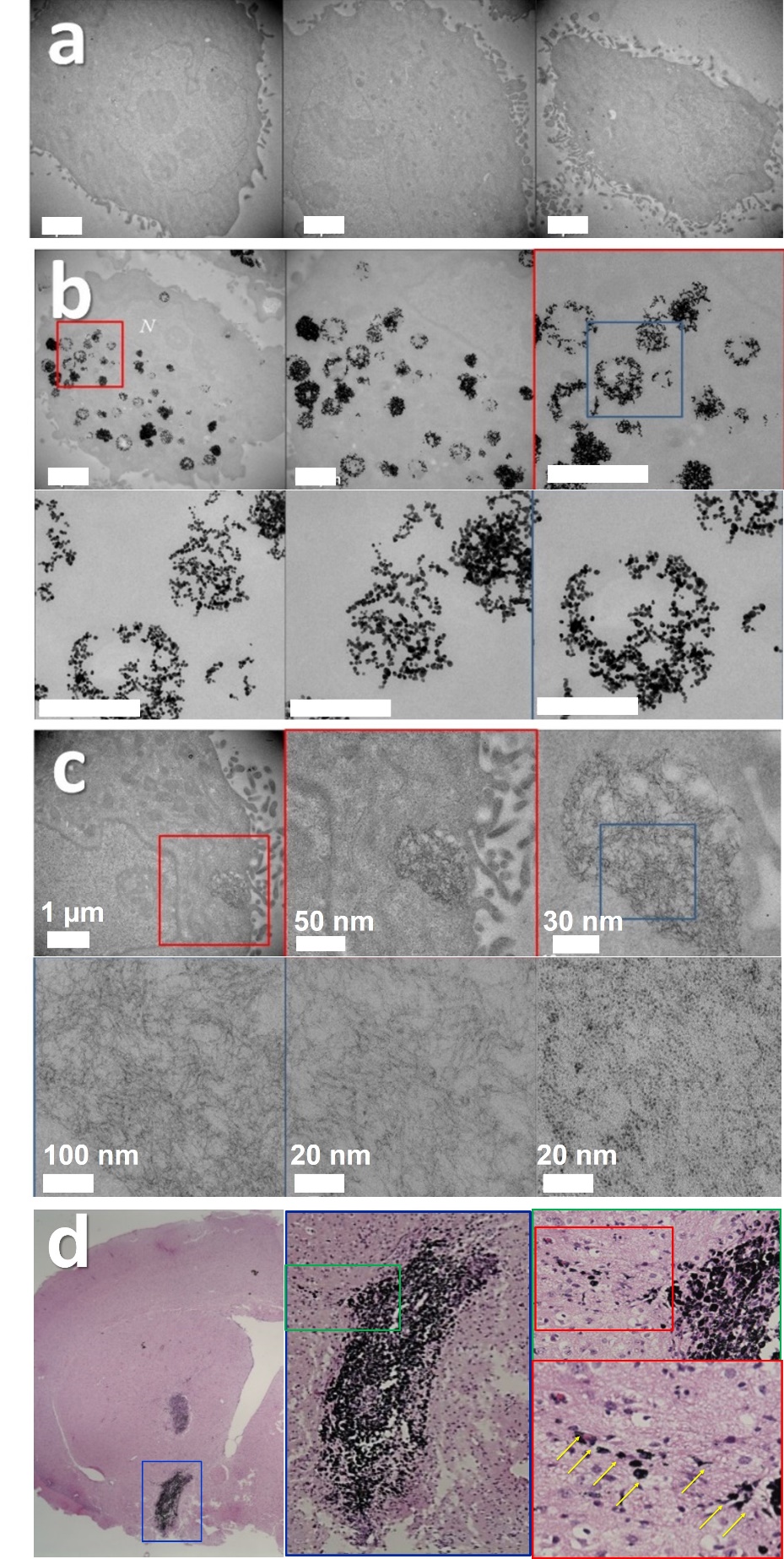


**Figur S2 -** Transmission electron microscope images of U87 cell in different conditions. (a) Control specimen, without any treatment. (b) and (c) Bare AuNPs MUA-AuNPs co-cultured with cells. (d) *In vivo* cellular tracking U87 glioma cells loaded with bare AuNPs demonstrated invasive glioma cells in brain.


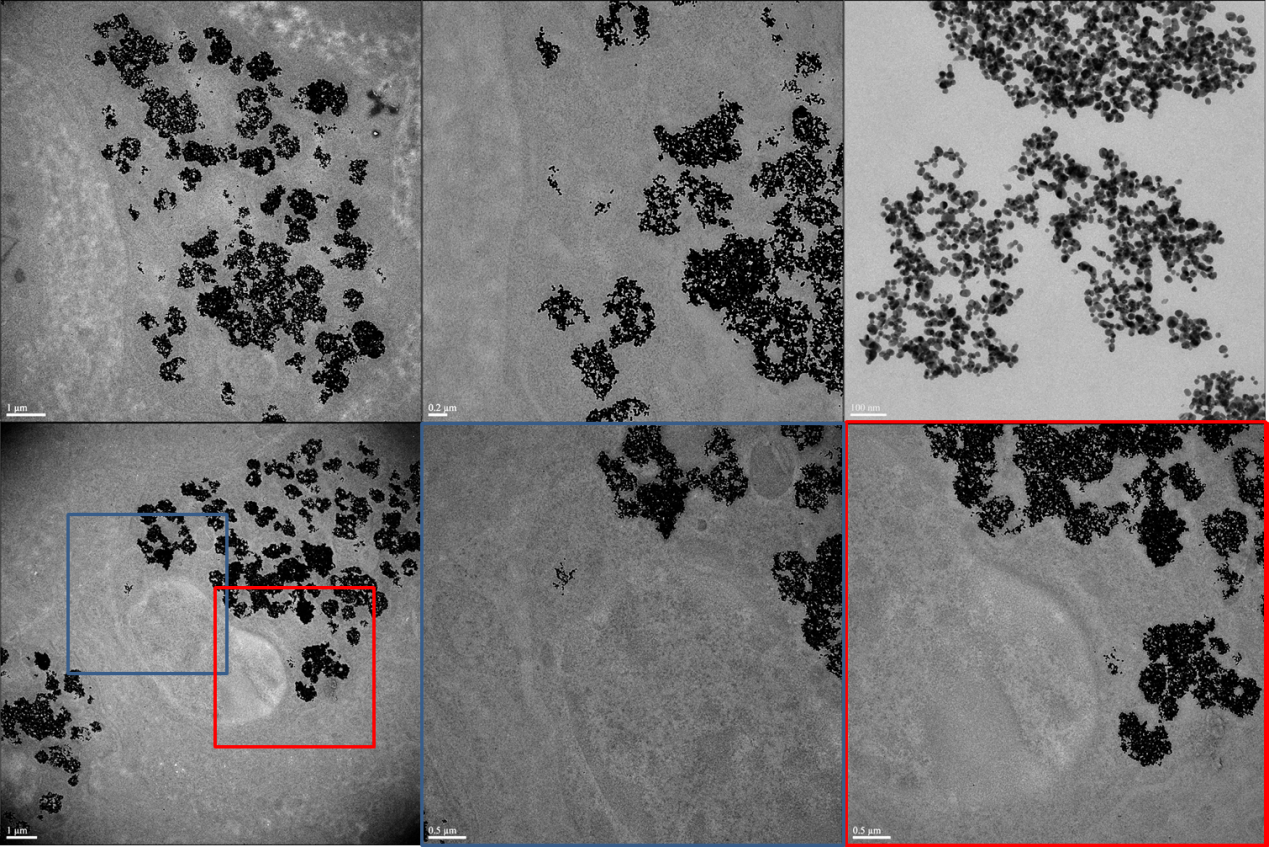


**Figure S3 -** Transmission electron microscope images of tumor cells with bare-AuNPs in brain tumor tissue. Scale bars: 1 µm.


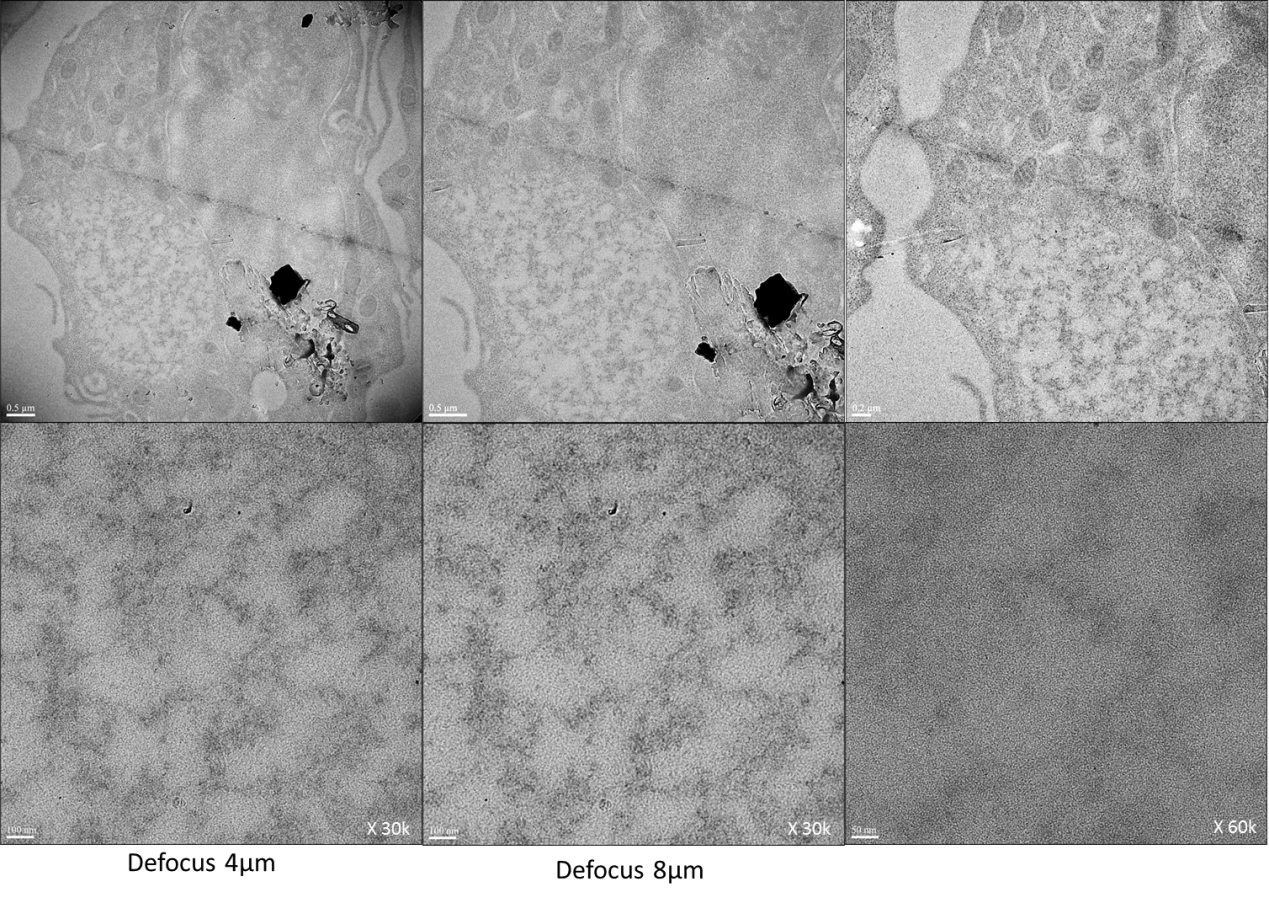


**Figure S4.** Transmission electron microscope images of tumor cells with MUA-AuNPs in brain tumor tissue. Scale bars: 0.5 µm.
